# Establishing a novel inflammatory bowel disease prediction model based on gene markers identified from single nucleotide variants of the intestinal microbiota

**DOI:** 10.1002/imt2.40

**Published:** 2022-07-24

**Authors:** Shuaiming Jiang, Denghui Chen, Chenchen Ma, Huanwei Liu, Shi Huang, Jiachao Zhang

**Affiliations:** ^1^ College of Food Science and Engineering Hainan University Haikou China; ^2^ Key Laboratory of Food Nutrition and Functional Food of Hainan Province Haikou China; ^3^ Department of Psychiatry University of California San Diego California USA; ^4^ Faculty of Dentistry The University of Hong Kong Hong Kong SAR China

**Keywords:** diagnostic markers, inflammatory bowel disease, intestinal microbiota, metagenome, single nucleotide variants

## Abstract

The intestinal microbiota is a crucial environmental factor in the development of inflammatory bowel disease (IBD). The abundance of *Faecalibacterium prausnitzii* is significantly decreased in IBD patients, which is used as a biomarker for IBD diagnosis. However, this can be observed in both IBD and colorectal cancer, which would confound the diagnostic results. Thus, we first established a new model for predicting Crohn's disease (CD) with high precision according to gene characteristics based on single nucleotide variants (SNVs). Next, five gene markers belonging to two species, *F. prausnitzii* and *Eubacterium rectale*, that were enriched in the CD group were obtained to build a CD prediction model, and high accuracy in distinguishing the CD and control groups was observed in the discovery (area under curve [AUC] = 91.13%) and validation cohorts (AUC = 79.55%). The model still maintained high accuracy after expanding the healthy cohort (AUC = 89.75%). High disease specificity in distinguishing CD and CRC groups (AUC = 95.74%) was also proven. This study establishes a novel diagnostic method for predicting IBD that also provides unprecedented insight for the early, painless diagnosis of other non‐communicable diseases.

## Highlights

• Specific gene characteristics based on single nucleotide variants were identified in intestinal microbiome of inflammatory bowel disease (IBD) patients.
• A novel and accurate diagnostic method for predicting IBD was established.
• *Faecalibacterium prausnitzii* showed a decreased abundance in different diseases and carried disease‐specific mutation sites within genes.

## Graphical abstract

Specific gene characteristics based on single nucleotide variants were identified in intestinal microbiota genes of inflammatory bowel disease (IBD) patients. A novel and accurate diagnostic method for predicting IBD was established. *Faecalibacterium prausnitzii* showed a decreased abundance in different diseases and carried disease‐specific mutation sites within genes.

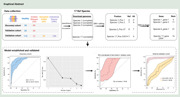

## INTRODUCTION

Inflammatory bowel disease (IBD) is a group of chronic immune‐mediated inflammatory diseases, including ulcerative colitis (UC) and Crohn's disease (CD), that are induced by alterations in the interaction between the intestinal microbiota and the intestinal immune system [1, 2, 3]. It has been found that the abundance of *Faecalibacterium prausnitzii* is significantly decreased in patients with IBD, which serves as a biomarker for IBD diagnosis [4]. However, decreases in *F. prausnitzii* occur not only in patients with IBD but also in patients with inflammatory other diseases, such as type 2 diabetes [5], colorectal cancer, and psoriasis [6]. In addition, the strains belonging to the same species may differ genetically by 5%–30% (or more) [7]. Thus, an IBD diagnosis based on species‐level bacterial abundance could lead to very questionable results. Recent research has shown that it is reliable to establish a prediction model based on single nucleotide variants (SNVs) to distinguish between colorectal cancer (CRC) and healthy cohorts [8]. Additionally, to reduce the effect of the sparsity of SNVs in intestinal microorganisms, normalizing SNVs at the gene level with annotation provides results more closely in line with the biological characteristics of adaptive evolution.

Here, we propose a new perspective on IBD pathogenesis based on the analysis of SNVs and the corresponding annotated genes of intestinal microorganisms. First, we carefully selected strains with sequencing depths higher than 10× and downloaded the whole genomes of standard strains from the National Center for Biotechnology Information (NCBI) database. Then, we screened intestinal microbial SNVs and calculated the total number of SNVs that belonged to each gene in each strain in the discovery cohort, which included CD (*n* = 68) and control groups (*n* = 34) [3]. Based on the gene characteristics of the SNVs, we developed a model for predicting CD with high precision. The verification of the model was carried out with a validation cohort including CD (*n* = 20) and control groups (*n* = 22). Furthermore, a UC group (*n* = 76) and another CRC disease group (*n* = 126) were used to test the specificity of the CD gene markers [8]. This study introduces a new diagnostic method for predicting IBD that allows a deeper assessment of the role of the intestinal microbiota in the course of disease and provides unprecedented insight into the early painless diagnosis of other non‐communicable diseases.

## RESULTS

### SNV calling and the construction and validation of gene markers in an IBD model

**Figure 1:**
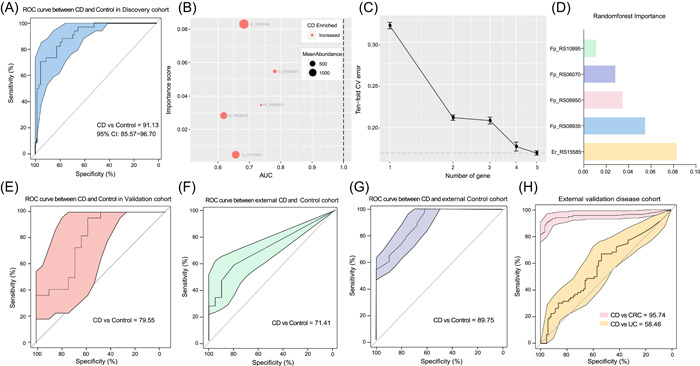
Model prediction and verification according to gene markers based on SNVs. (A) The prediction results in the discovery cohort with an accuracy of 91.13% between CD and control samples. (B) The importance scores and mean abundances of the five selected gene markers are shown. (C) The 10‐fold cross validation error plot of the five selected genes. (D) The random forest importance scores of the five selected genes. (E) The prediction results from the validation cohort between CD and control samples. (F) The ROC curve between external CD and healthy samples. (G) The prediction results between CD (*n* = 68) and external control samples (*n* = 112). (H) The distinct characteristics of the five gene markers used to classify CD and other diseases. Orange represents the ROC curve between the CD and UC groups, and pink represents the accuracy of the gene model in classifying the CD and CRC groups. CD, Crohn's disease; CRC, colorectal cancer; ROC, receiver operator characteristic; SNV, single nucleotide variant; UC, ulcerative colitis.

We used the whole genomes of 17 strains to build the genome library, and the metagenome of each sample was compared with the library for SNV calling. The samples belonging to project PRJNA400072 were divided into a discovery cohort (*n* = 102) and a validation cohort (*n* = 42). For the discovery cohort (*n* = 102), including 68 CD patients and 34 healthy controls, a total of 558,738 nonredundant SNVs were annotated to 28,816 genes. The location and number of SNVs in a gene may directly affect the function of the gene and in turn affect the evolution of microorganisms. Taking the number of SNVs on 28,816 genes as characteristics, the random forest modeling was carried out, and the importance scores of different gene characteristics were obtained and sorted. After comparing the numbers of SNVs in genes in the CD and control groups, the five genes with importance scores greater than 0.01 were selected based on the random forest result for the discovery cohort (Supporting Information: Table S3). The 10‐fold cross‐validation of different numbers of selected genes was conducted on the discovery cohort data with an error rate lower than 0.175 when using the five selected genes (Figure 1C). Finally, a CD prediction model based on the five selected gene markers and the random forest algorithm was built. The corresponding importance scores for each gene in the final model are shown in Figure 1D. The five selected gene markers, gene‐C4Q21_RS08950 (Fp_RS08950), gene‐C4Q21_RS08935 (Fp_RS08935), gene‐C4Q21_RS06070 (Fp_RS06070), gene‐C4Q21_RS10895 (Fp_RS10895), and gene‐EUBREC_RS1558 (Er_RS15585), belonged to two species, *F. prausnitzii* and *Eubacterium rectale*, as shown in Supporting Information: Table S4. The specific SNV mutation information for each gene is shown in Supporting Information: Table S5. In the discovery cohort, the accuracy of distinguishing the CD and control groups was 91.13% (Figure 1A), and five gene markers were all enriched in the CD group according to the Wilcoxon rank‐sum test (Figure 1B).

In the validation cohort (CD: *n* = 20, control: *n* = 22), the area under curve [AUC] value also reached 79.55% (Figure 1E). Other public IBD datasets applied for secondary verification also support the accuracy of gene markers, and AUC value between CD and Control samples reached 71.41% (Figure 1F). In contrast the accuracy of using the prediction model based on the five gene markers to distinguish between the UC (*n* = 76) and CD (*n* = 88) groups reached only 58.46% (Figure 1H), which indicated that in IBD patients, both UC and CD were chronic inflammatory reactions that occurred in the intestinal tract and that there were overlapping or similar mutation sites in individuals with both diseases. The model specificity was be verified between CD samples in discovery cohort (*n* = 68) and external healthy people cohort (*n* = 112), with a high accuracy of 89.75%, as shown in Figure 1G. Moreover, due to the increased risk of CRC in patients with IBD, the CRC cohort (*n* = 126) was also used to verify the specificity and accuracy of these gene markers in different intestinal diseases, and the CD gene markers could be used to separate the CD (*n* = 88) and CRC (*n* = 126) groups with a high accuracy of 95.74%, as shown in Figure 1H.

### Functional annotations related to five CD‐enriched genes

**Figure 2:**
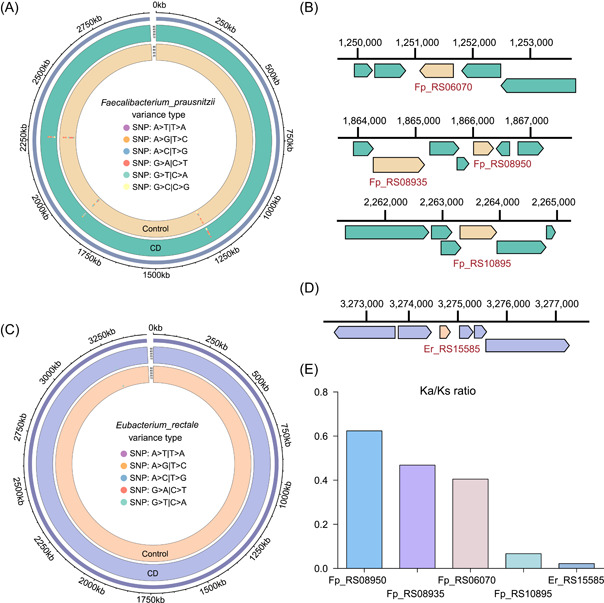
Mutation‐based functional annotation of five gene markers. (A, B) The mutation types and locations of the SNVs in *Faecalibacterium prausnitzii*; (C, D) the mutation types and the locations of the SNVs in *Eubacterium rectale*; (E) Ka/Ks is the ratio of the nonsynonymous substitution rate (Ka) to the synonymous substitution rate (Ks). A Ka/Ks ratio of 1 indicates that the studied genes evolved under neutral selection; a value of less than 1 indicates evolution under purifying selection; and ratio greater than 1 is considered to indicate evolution under positive selection. SNV, single nucleotide variant.

Among the five gene markers, four genes, including Fp_RS08950, Fp_RS08935, Fp_RS06070, and Fp_RS10895, belonged to *F. prausnitzii*, and one gene, Er_RS15585, belonged to *E. rectale*. The mutation types and the locations of the SNVs in *F. prausnitzii* and *E. rectale* are shown in Figure 2A–D, where different colored points represent different mutation types, and the locations of the points indicate where the mutations occur in the genome. The outer circle represents the CD group, and the inner circle represents the control group. The Er_RS15585 gene functions as a helix‐turn‐helix domain‐containing protein (WP_003505382.1), and the Fp_RS08950 gene was functionally annotated as a hypothetical protein (AXB29042.1). The Fp_RS08935 gene can express a zf‐HC2 domain‐containing protein (AXB29039.1), the Fp_RS06070 gene can produce peptidase S51 (AXB28508.1), and the Fp_RS10895 gene functions as a replication protein (AXB29383.1). The Ka/Ks ratios of the five genes are shown in Figure 2E. The Ka/Ks ratios of Fp_RS08950, Fp_RS08935, and Fp_RS06070 were between 0.4 and 0.6, while the Ka/Ks values of Fp_RS10895 and Er_RS15585 were much less than 0.1, which indicated that they were all under purifying selection. This may be because general nonsynonymous substitution results in harmful traits and will lead to evolutionary advantages in only a few cases [9, 10]. The finding that the mutations in these genes were under purifying selection indicated that the genes were being eliminated.

## DISCUSSION

*F. prausnitzii* and *E. rectale*, which belong to Firmicutes, can readily ferment soluble fiber to produce short‐chain fatty acids (SCFAs) in the intestinal tract [11], and SCFAs are considered to have numerous benefits, including increasing the levels of anti‐inflammatory cytokines and preventing inflammatory diseases [12]. In this study, four of the selected gene markers belonged to *F. prausnitzii*. Among them, the Fp_RS06070 gene was functionally annotated as peptidase S51, which is believed to have a nutritional function [13]. It is speculated that under the conditions of the limited intestinal niches present in IBD patients, the abundance of *F. prausnitzii* decreases, and more mutations related to nutrient uptake occurred to allow it to survive. Additionally, *F. prausnitzii* produces butyrate to maintain the balance of the intestine, enhance intestinal immunity, and subsequently affect the course of IBD [14]. Moreover, the Fp_RS08935 gene can produce a zf‐HC2 domain‐containing protein. Interestingly, in the CRC cohort, SNV markers were also enriched in the Fp_RS08935 gene [8], which may be due to more similar intestinal conditions in CD and CRC patients, but the specific mutation sites and numbers were different. These findings proved that it is feasible to establish prediction models in different disease cohorts based on SNVs, especially when the strain abundance changes in similar ways.

Additionally, mutations were enriched in the CD group, indicating that in the case of niche limitation in the disease group, more mutations were more likely to occur. Five gene markers can be used for gene amplification for the painless and rapid advance prediction of CD. This study establishes a novel diagnostic method for predicting IBD that allows a deeper interpretation of the role of the intestinal microbiota in the course of disease and provides unprecedented insight for the early, painless diagnosis of other diseases.

## CONCLUSION

By collecting the shotgun metagenomic data of intestinal microbiota in IBD patients and healthy people, the SNVs in intestinal microbiota genes were used as characteristics for screening potential biomarkers. Final, five specific gene characteristics based on SNVs were identified in intestinal microbiota genes of IBD patients. The predictive model based on the five gene markers had high accuracy in distinguishing the CD and control groups, which made a deeper interpretation of the role of intestinal microbiota in the course of disease, and provided unprecedented insight into the early painless diagnosis of other diseases.

## METHODS

### Sequence data collection

Shotgun metagenomic data from fecal samples of human IBD patients and healthy people were collected. The raw data were downloaded from NCBI, and the specific characteristics of the sequencing data can be found in Supporting Information: Table S1. According to the original data classification in PRJNA400072 [3], the data were divided into discovery and validation cohorts. A total of 68 IBD patients and 34 healthy controls (SRA accession number: PRJNA400072 [3]) were used for modeling as the discovery cohort, and 22 healthy controls and 20 CD patients were used for validation. We also collected public IBD datasets for secondary verification, including datasets PRJNA398089 [15] and ERP002061 [16]. Additionally, 76 UC samples (SRA accession number: PRJNA400072 [3]) and 126 colorectal cancer (CRC) samples were employed to verify the specificity of the gene markers (SRA accession numbers: ERP008729 [17], DRA006684 [18], PRJNA663646 [8], and SRP136711 [19]). All datasets used in this study can be found in Table 1.

**Table 1:** Fecal metagenomic studies included in this meta‐analysis

Data set	Disease related	Sampling niche	Library strategy	Sequencing platform	Number of cases	Number of controls	Host geolocation	Data source
PRJNA400072	Inflammatory bowel disease, Crohn's disease	Human faeces	WGS	ILLUMINA	88	56	Boston, Netherlands	PRJNA400072
PRJNA398089	Inflammatory bowel disease, Crohn's disease	Human faeces	WGS	ILLUMINA	2	6	American	PRJNA398089
ERP002061	Inflammatory bowel disease, Crohn's disease	Human faeces	WGS	ILLUMINA	13	106	Danish, Spanish	ERP002061
PRJNA400072	Inflammatory bowel disease, ulcerative colitis	Human faeces	WGS	ILLUMINA	76	N/A	Boston, Netherlands	PRJNA400072
ERP008729	Colorectal cancer	Human faeces	WGS	ILLUMINA	46	N/A	Austria	ERP008729
PRJNA663646	Colorectal cancer	Human faeces	WGS	ILLUMINA	8	N/A	Hainan, China	PRJNA663646
SRP136711	Colorectal cancer	Human faeces	WGS	ILLUMINA	32	N/A	Italy	SRP136711
DRA006684	Colorectal cancer	Human faeces	WGS	ILLUMINA	40	N/A	Japan	DRA006684

### Identification of microbial taxonomy and SNV calling

Due to the limitation of SNV annotation imposed by the depth and coverage of metagenomic sequencing, we annotated microbial species using MetaPhlan2 [20] and selected the strains with an average relative abundance greater than 0.5% for SNV annotation to ensure high quality. The information on all 17 selected reference genomes, representative strains from the NCBI database and their GenBank accession numbers is listed in Supporting Information: Table S2. Subsequently, the metagenomic sequencing reads were mapped to the reference genomes for SNV calling by using SAMtools (v1.11) and BCFtools (v1.8) [21]. Thereafter, the total number of SNVs belonging to each gene in each strain was calculated. Since the relative abundance of strains directly affected the number of SNVs annotated, we also standardized the SNVs in a gene by dividing the SNV count in the gene by the relative abundance of the strain and the depth of sequencing. More details of the code can be found on GitHub (https://github.com/jsming1996/IBD_project).

### Statistics statement and modeling

The number of annotated SNVs in the genes was used as the parameter for screening potential biomarkers using the “randomForest” package in R [22]. The enrichment of genes between different groups was calculated by the Wilcoxon rank‐sum test (*p* < 0.05) [23]. Receiver operator characteristic (ROC) analysis to evaluate the accuracy of the SNV biomarkers was performed with the “pROC” package in R [24]. The genomic Circos plot used to annotate the locations of the genes was drawn with the “circlize” package [25] and “grid” in R. The Ka/Ks ratio, representing the ratio of nonsynonymous mutations to synonymous mutations, was calculated using KaKs_Calculator 2.0 [26].

## AUTHOR CONTRIBUTIONS

The study was designed by Jiachao Zhang. Data collection was performed by Shuaiming Jiang, Huanwei Liu, and Chenchen Ma. Data analysis was performed by Shuaiming Jiang, Denghui Chen, and Shi Huang. The manuscript was written by Shuaiming Jiang. All authors read and approved the final manuscript.

## CONFLICT OF INTEREST

The authors declare no conflict of interest.

## Supporting information

Supporting information.

## Data Availability

The data are available in a public database (National Center for Biotechnology Information) under accession numbers PRJNA400072, PRJNA398089, ERP002061, ERA000116, ERP008729, DRA006684, SRP136711, and PRJNA663646 (metagenome sequencing). The scripts used are saved in GitHub (https://github.com/jsming1996/IBD_project). Supporting Information (figures, tables, scripts, graphical abstract, slides, videos, Chinese translated version and update materials) may be found in the online DOI or iMeta Science http://www.imeta.science/.
